# Regulatory Approval With Real-World Data From Regulatory Science Perspective in Japan

**DOI:** 10.3389/fmed.2022.864960

**Published:** 2022-04-15

**Authors:** Hideki Maeda, Daniel Bin Ng

**Affiliations:** ^1^Department of Regulatory Science, Faculty of Pharmaceutical Sciences, Meiji Pharmaceutical University, Kiyose, Japan; ^2^Department of Medical Affairs, Astellas Pharma, Singapore, Singapore; ^3^Department of Pharmacy Systems, Outcomes and Policy, University of Illinois at Chicago College of Pharmacy, Chicago, IL, United States

**Keywords:** real-world data (RWD), real-world evidence (RWE), regulation, approval, registry

## Abstract

Recently, there has been a growing trend in clinical development to utilize real-world data (RWD) to improve the efficiency of drug/medical device development. Especially, the use of RWD to generate real-world evidence (RWE) in regulatory approval is currently undergoing a period of great change with an increasing degree of active discussion. In Japan, RWE has been used in the control arms of clinical trials, observational studies, post-marketing surveillance, and public knowledge-based applications for regulatory approval. However, the exclusive use of RWE applications has still not been applied. In this paper, we summarize the history and the current situation of RWE and focus on the utilization for the purpose of regulatory approval. In addition, we will discuss the issues and perspectives for registry research in the utilization for regulatory approval in Japan.

## Introduction

Recently, the use of large-scale real-world data (RWD) has been increasing, and as a result, its application has also been expanding. In the field of regulation of drugs and medical devices, utilization of RWD has been actively discussed and developed rapidly. In particular, the utilization of real-world evidence (RWE) for pharmaceutical approval is a big topic across the industry. In all stages of development, review, approval, and commercialization, discussions have been held on the utilization of RWD, such as electronic health records (EHR), claims data, pharmacy data, and diagnosis procedure combination (DPC) data, with high expectations for their implementation. Japan and Asia are no exception (1, 2).

However, the development of legislation/guidelines/rules on the use of RWE in pharmaceutical regulation are still developing. In Japan, the legislation of clinical research is established and well-defined. “Ethical Guidelines for Medical Research Involving Human Subjects” (3) and “Ethical Guidelines for Human Genome/Analysis Research” (4) were integrated, and released on March 23, 2021, as new set of ethical guidelines (Ethical Guidelines for Medical and Biological Research Involving Humans Subjects) (5). Most RWE research, which often use observational clinical studies, follow this new ethical guideline. Japan Pharmaceutical Manufacturers Association (JPMA) has also released various guidance and white papers on the use of RWE (6), and has been involved in several activities aimed at implementing RWE associated with approval for pharmaceuticals, with a particular focus on utilizing registry data. In April 2018, the “Amendment of Regulation for Good Post Study Practice for Pharmaceutical Affairs and Medical Devices [Revised Good Post-Marketing Study Practice (GPSP)]” was implemented which was issued to clarify clinical questions and carry out post-marketing surveillance based on clinical questions. It stated that RWD can now be used for post-marketing surveillance by the Revised GPSP (7). Currently, Medical Information Database Network (MID-NET) is available to conduct such research (8, 9).

When considering using RWE for pharmaceutical approvals, one of the most important issues is the reliability of data. Historically, in Japan, the standards and types of EHR and pharmacy data have been developed separately resulting in non-unified standards and inconsistencies which potentially makes this data source unsuitable to use for drug approval (10). Furthermore, when considering the reliability, the type, the granularity, the scope, and the traceability of the data, registry data is often seen as easy to handle, unified, and adapted for pharmaceutical approvals (11, 12).

The objective of this study is to summarizes the current situation regarding the utilization of RWD in Japan, the movement of the regulatory authorities, and the actions of healthcare industries. The study also aims to clarify the current status, implementation methods, and points to keep in mind for future registration research by using registries as a typical case of RWE.

### Current Status of the Use of RWD

#### Situations of the Utilization of RWD in Development and Regulatory Approval for Drugs/Medical Devices

There are several possibilities for the use of RWD in clinical development, especially in the regulatory approvals (13). The use of RWD in clinical data package of application for regulatory approvals are historical control group, it is also possible to use as part of the clinical data packages involving special patient populations such as children, the elderly, and patients with renal and/or liver disfunctions. It could also be use of a broaden population associated with conditional approval. In addition, an effective use of RWD could identification and in response to a safety signal as well as in Post-Marketing Requirements (PMR) and Post-Marketing Commission (PMC). In Japan, the 2018 revision of the GPSP legally approved the use of RWD in Post-Marketing Surveillance. RWD is often used for public knowledge-based applications, which are a unique application of public information (14).

#### Examples of Drug Approval Using RWD in the United States and Europe

Examples of regulatory approvals using RWD in the United States and Europe are shown in Table 1. There are nine cases in the United States and/or Europe including palbociclib (15–17). Where the FDA granted the additional indication for male breast cancer in April 2019 without additional clinical studies. In the application, only three RWDs (IQVIA claims data, Flatiron Health ER data, Pfizer global safety data) were used to obtain approval (18). This is the first case where additional indications were granted without the need for a positive clinical test. On the other hand, for selinexor and erdafitinib, the authorization application included RWE, but it was judged by FDA as insufficient evidence due to a mis-matched the patient background and starting condition of the outcome, and the unified effect evaluation standard was not used.

**Table 1:** Approval applications using real world data in Japan, US and Europe.

**Generic name**	**Indication**	**Japan**			**US**			**Europe**		
		**Approval year**	**iNDA/sNDA**	**Data source**	**Approval year**	**iNDA/sNDA**	**Data source**	**Approval year**	**iNDA/sNDA**	**Data source**
Algucosidase alfa	Pompe disease	2007	iNDA	External control medical records (overseas)	2006	iNDA	External control medical records	2006	iNDA	external control medical records
Argatroban	Heparin-induced thrombocytopenia	2011	sNDA	External control medical records (overseas)	–	–				
Methotrexate	Rheumatoid arthritis	2011	sNDA	Public-knowledge application post-marketing surveillance	–	–				
Tacrolimus	Interstitial pneumonitis in polymyositis/ dermatomyositis	2013	sNDA	External control published article (Japanese)	–	–				
Methylprednisolone Sodium Succinate	Multiple sclerosis	2013	sNDA	Public-knowledge application post-marketing surveillance	–	–				
Asfotase Alfa	Hypophosphatasia	2015	sNDA	External control electric health record (overseas) registry (overseas)	–	–				
Avelumab	Merkel cell carcinoma	–	2017	iNDA	External control electric health record published article	2017	sNDA	External control electric health record registry		
Cerliponase alfa	Neuronal ceroid-lipofuscinosis	–	2017	iNDA	External control registry	2017	iNDA	External control registry		
Tisagenleceucel	B-cell acute lymphoblastic leukemia	–	–			2018	iNDA	Observational study registry		
Paliperidone Palmitate	Schizophrenia	–	2018	Revise labeling	Pragmatic clinical trial	–				
Palbociclib	Male breast cancer	–	2019	sNDA	Observational study electric health record claim data adverse events database	–			
Selinexor	Multiple myeloma	–	2019	iNDA	External control electric health record	–				
Erdafitinib	Urothelial carcinoma	–	2019	iNDA	External control electric health record	–				
Tacrolimus	Prevent organ rejection receiving lung transplantation	–	2021	sNDA	Observational study external control registry	–				

#### Examples of RWD's Use in Regulatory Approval in Japan

Examples of approval applications using RWE in Japan are also shown in Table 1. There are six cases in Japan (19). For Algucosidase alfa, Argatroban, and Asfotase Alfa, data collected from overseas medical records and EHR or registries were compared with data extracted from published papers. In the two cases of public knowledge-based application, the registration and the actual usage survey results were used. However, there have been no examples of approval using Japanese RWD.

#### Regulations and Rules Related to RWD in Japan and the US/Europe

**Figure 1:**
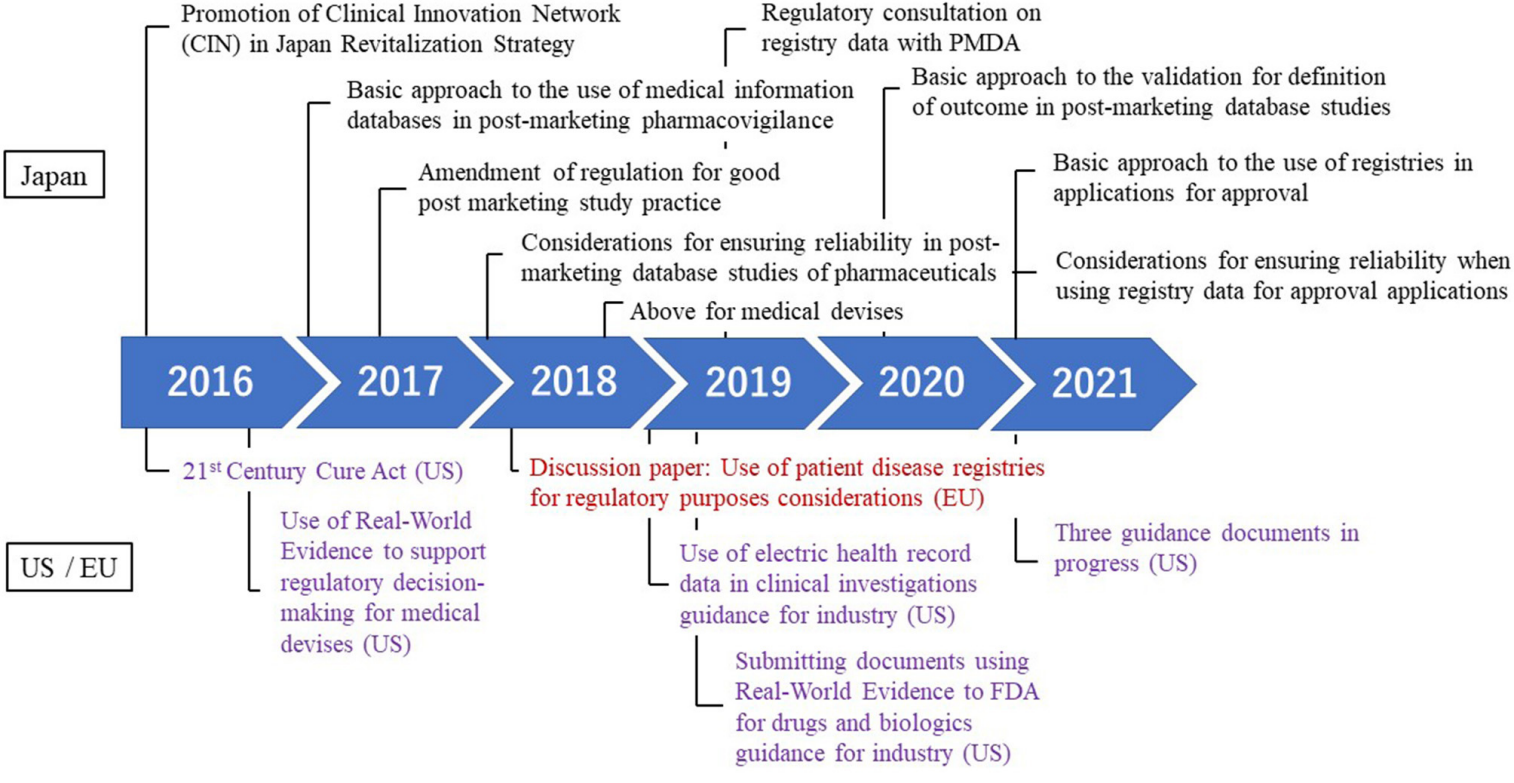
Regulatory developments related to real world data.

Regulatory developments related RWD in Japan and the US and Europe is shown in Figure 1. In recent years, discussions to utilize patient registries under the pharmaceutical system have been actively conducted in Japan Agency for Medical Research and Development (AMED) (20), regulatory authority (21), pharmaceutical industries (6), and the establishment of a Clinical Innovation Network (CIN).

Looking back on the development in Japan, in June 2016, while discussing the utilization of RWD in the Japan Revitalization Strategy, the decision was made to promote the establishment of the CIN and to create an advocacy system. Subsequently, in Japan, the utilization of the clinical database study, particularly in post-marketing surveillance, was actively discussed. In June 2017, the “Basic approach to the use of medical information databases in post-marketing pharmacovigilance” was issued (22). Following this, the GPSP was revised in April 2018 and the database research became available for commercial research (7). In addition, with a view to the time when the reexamination application was made, a notification was issued in February 2018 entitled “Considerations for ensuring reliability in post-marketing database studies of pharmaceuticals” (23). Currently, post-marketing research are conducted using databases that often includes the MID-NET recommended by PMDA. Insights often point to PMDA continuing to make efforts to promote the appropriate utilization of RWD/RWE in the regulatory setting (24).

The utilization of RWE in view of the approval of pharmaceutical and medical devices is a worldwide movement, and the development of the legislation has advanced in the US. First, in the US, the use of RWD for regulatory decisions began to be considered with the passage of the 21st Century Cures Act (25). And then, in August 2017, the US FDA issued “Use of Real-World Evidence to Support Regulatory Decision-Making for Medical Devices” to recommend the use of RWE in medical device development as early as possible (26). In the following year, the US government announced that it was considering using RWE in regulatory evaluation to determine the effectiveness of pharmaceutical products. In July 2018, the “Use of Electronic Health Record Data in Clinical Investigations” guidance on the use of EHR data for pharmaceutical approval was issued (27). In October 2021, a draft guidance was issued for the data standards for drug and biological production submission utilizing RWE. And the US FDA released the commitment letter outlining performance goals and procedures for the upcoming reauthorization of the Prescription Drug User Fee Amendments for Fiscal Years 2023–2027 (PDUFA VII) (28), and it is expected that the use of RWE will be further advanced. For European regulators, a discussion paper on registration was prepared in 2018 (29) and a draft of guidance was prepared in September 2020 (30). In February 2022, EMA established the Coordination Centre for the Data Analysis and Real World Interrogation Network, and EMA will maintain a real-world data sources and metadata for use in medicine regulatory activities (31).

### Registry Research

As already discussed, considering the reliability, scope, and traceability of the data, the use of a registry data is reasonable and is one of the best RWD to be adapted for regulatory approval. In this section, we describe the overview of registry studies and the current status in academia and pharmaceutical industry.

#### Overview

Registry research is of the application of observational research and non-interventional study. The purpose of the registry research is divided into two aspects. One is to collect various data on the number and distribution of patients, the treatment of diseases, and their effectiveness and safety to help improve the understanding of diseases and medical care. As concrete examples, national cancer registration and cohort research were, so far, the most common types of registry research in Japan. The second objective of registry research is for new drug clinical development and safety measures in post-marketing situations.

In Japan, the movement to utilize highly reliable registry data for drug approval rather than using RWD such as EHR and claims data for drug approval is more prevalent. In April 2019, a registry consultation was established at PMDA, to begin the examination of registry research of specific pharmaceutical and medical devices. The guidance was issued in March 2021, with ”Basic approach to the use of registries in applications for approval“ (32) and ”Considerations for ensuring reliability when using registry data for approval applications“ (33).

Registry research is assumed to have a slightly different set of rules depending on whether it is for academia or the private industry. However, there is no significant difference between registry research whether it is led by a the pharmaceutical company or academia, and following the ”Ethical Guidelines for Medical and Biological Research Involving Human Subjects“ which refers to the Next Generation Medical Infrastructure Law (enforced on 11 May 2018) for the maintenance of anonymity in the registry date (34). Regarding company-leading research, it is necessary to follow the Act on the Protection of Personal Information (enforced in 2005) for patient information (35).

#### Movement in Academia

The academia movement on registry research includes a project led by the Ministry of Health, Labour, and Welfare to support the promotion of the Clinical Innovation Network (CIN). The CIN promotion support project consists of three research projects. These research projects exist as a cross-sectional research team that examines various, related issues. They include ”Research on Promotion Measures for Clinical Innovation Network Concept by Effective Use of Disease Registry Systems,“ which was started in 2015 to effectively utilize fund and resolve ethical issues, ”Basic approach to ensuring ethics in the corporate use of patient registry data“ (36) and ”Study on the cost burden for the utilization of disease registration systems“ (37).

In addition, there are also discussions on the construction of disease-specific registries such as the field of muscular dystrophy, amyotrophic lateral sclerosis (ALS), a rare fraction of cancer, and neurosurgery diseases. These CINs are currently developing the basis for registries and research to increase the reliability of the data.

#### Movement in Pharmaceutical Industries

Since disease registries contain clinical information and treatment results specific to that disease, it is considered an invaluable source of insights. It's ability to capture relevant data often used in clinical practice results in healthcare industries paying particular attention to this data source. In particular, the JPMA has been carrying out activities related to disease registries in its subordinate organization, the Clinical Evaluation Subcommittee of JPMA (CES-JPMA), and has been involved in various projects. Since 2017, using eye disease registries, the CES-JPMA analyzed policy trends in Japan and abroad, including disease-specific applications, current status surveys of typical registries, proposals for ecosystem formation, and issues to be used for approval applications. Starting around 2019, CES-JPMA has been compiling examples of actual applications, considering how to use them for pharmaceutical applications, and conducting surveys on registries. At present, the outputs and white papers that serve as indicators for the utilization of pharmaceutical applications for approval are being released (6).

#### Ethical Issues on Industry Use for Registry Research and RWD

Various kinds of big data and RWD can be utilized by healthcare industries (Table 2). Registry data is also expected to be a common source of big data which can be utilized for various pharmaceutical regulations in the future. However, there are some ethical issues for industry use of patient registry data in terms of personal information in Japan.

**Table 2:** Types of real world data and pros/cons for utilization by medical companies.

	**Health care data**	**Electronic health records**	**Disease registry**
Source data	- Claim data - Receipt data - DPC data (diagnosis procedure combination) - Accounting data	- Electric health records - Nursing records - Medical order records	- Registry data - Observational data - Daily practice data
Advantages	- Many patients - Easy to standardize - Easy to structuring	- Reflecting daily practice - Many items	- Obtain data that are not recorded in daily practice.
Disadvantages	- There can be an insurance disease name (other than the real disease name).	- Many non-structured data - Different operations and standards depend on institutions	- Large burden of work on data collection

After the amendment of the Act on the Protection of Personal Information (35) in Japan, medical information on specific individuals, including those captured in registries, was categorized as ”special care-requested personal information“ in Japan. In addition, according to ”Ethical Guidelines for Medical and Biological Research Involving Human Subjects“, when a researcher tries to obtain and utilize a ”special care-requested personal information,“ it stated that ”researchers and others do not necessarily have to receive an informed consent, but if they do not receive an informed consent, they have to receive an appropriate consent from the patients". Therefore, the use of opt-out method was a practical method applied in these situations. However, when the use of the opt-out system is intended for purposes other than academic research (e.g., industry use), there are some that argue that the opt-out method may be ethically inappropriate (38).

Regarding clinical research based on Article 68 of the Pharmaceutical and Medical Device Act, since the informed consent for providing personal information to the healthcare company does not need use of pharmaceutical products, there are cases that the registries used by medical institutions can provide the data to the healthcare industries without consent based on the law. However, it is recommended that the scope of use personal information in this framework should be limited to regulatory purposes. Specifically, it should be limited to clinical research for the purpose of new drug application and described in Risk Management Plan (RMP).

## Discussion and Conclusion

In the US and Europe, discussions on the use of RWD by regulatory authorities have been progressing in recent years, and the number of cases in which companies submit RWE as a part of the data package for approval applications is increasing. In many cases, the common application focuses on rare diseases or conditions with an absence of other effective treatment. In recent years, there have been many cases in the US and Europe where data obtained from EHR, claim data, and registers are used to apply for approval as external control. In Japan, RWD has been used in the control arms of clinical trials, observational studies, post-marketing surveillance, and public knowledge-based applications for regulatory approval decision making. However, there are no cases of RWD-only application for regulatory approval in Japan. And there are also no examples in Japan where RWE based on EHR or Japanese registries have been used for regulatory approval. Although the public knowledge-based application is a form of approval application based on RWE, it takes a long time to apply for approval because it utilizes non-adaptation and accumulated data from clinical research. The application of RWE in the regulatory decision making for approval is still inadequate in Japan, and it is important to accumulate the results of the employment of RWD and it cases of its use in the regulatory decision.

## Author Contributions

All authors listed have made a substantial, direct, and intellectual contribution to the work and approved it for publication.

## Funding

The part of this study was granted by Research on Regulatory Science of Pharmaceuticals and Medical Devices, Health, Labour and Welfare Policy Research Grant No. 21KC2006.

## Conflict of Interest

DN is an employee of Astellas Pharma Global Development. The remaining author declares that the research was conducted without any commercial or financial relationships that could be construed as a potential conflict of interest.

## Publisher's Note

All claims expressed in this article are solely those of the authors and do not necessarily represent those of their affiliated organizations, or those of the publisher, the editors and the reviewers. Any product that may be evaluated in this article, or claim that may be made by its manufacturer, is not guaranteed or endorsed by the publisher.
